# Selecting a risk assessment tool to use in practice:a 10-point guide

**DOI:** 10.1136/eb-2017-102861

**Published:** 2018-04-27

**Authors:** Seena Fazel, Achim Wolf

**Affiliations:** 1 Department of Psychiatry, Warneford Hosptial, University of Oxford, Oxford, UK; 2 Research Department, St. Andrew’s Healthcare, Northampton, UK

## Abstract

With the increase in the number of risk assessment tools and clinical algorithms in many areas of science and medicine, this Perspective article provides an overview of research findings that can assist in informing the choice of an instrument for practical use. We take the example of violence risk assessment tools in criminal justice and forensic psychiatry, where there are more than 200 such instruments and their use is typically mandated. We outline 10 key questions that researchers, clinicians and other professionals should ask when deciding what tool to use, which are also relevant for public policy and commissioners of services. These questions are based on two elements: research underpinning the external validation, and derivation or development of a particular instrument. We also recommend some guidelines for reporting drawn from consensus guidelines for research in prognostic models.

Criminal justice has gradually started to evaluate treatments and interventions in more rigorous ways, including through conducting randomised controlled trials (RCTs). However, one area that has lagged behind is the area of risk assessment for violent outcomes, and, in particular, reoffending.^1^ Most high-income countries have some set of tools that are applied to assess risk of repeat offending and are used variously at sentencing, parole hearings, probation or on release from prison to determine aftercare. Over 200 of such tools exist and more appear every month.^2^ One recent example is a predictive tool being used in criminal justice in the US state of Georgia, which is based on five static items, and can be completed online.^3^ On the basis of these items, it provides a determination of low, medium or high risk without information about what these categories mean or any data on the accuracy of the tool. In addition, there are needs-based tools that are considerably more time consuming, and, to our knowledge, not been subject to RCTs to test their effects on outcomes. Overall, it is not known whether most of these risk instruments have been developed and validated according to evidence-based methods, partly as criminal justice agencies and those who work with them do not publish their findings in peer-reviewed journals, and the information provided in online reports is mostly limited to development work. To take two examples from the UK, the current approach entitled DASH to risk assess domestic violence is based on a model apparently developed by one individual without external validation.^4^ In criminal justice, a 100-point risk assessment tool in England and Wales is applied to all individuals receiving sentences of more than 12 months and has been adopted in many different countries. Although validated in England and Wales by the same team who developed the tool,^5^ which is an advance over many other instruments, we have not been able to identify one independent validation and none in samples outside of England and Wales.

With the increasing number of these instruments and the clear need to stratify individuals who pass through the criminal justice system on the basis of risk, we would like to propose an approach to evaluate the quality and underlying evidence base for a particular risk assessment. This should assist in reviewing existing approaches and in considering new instruments. To do this, we have drawn on methodological guidelines for prognostic research,^6^ systematic reviews of the risk assessment in criminal justice, and primary studies of the performance of these tools in real-world settings^7–9^(analogous to clinical effectiveness studies when investigating whether RCTs apply in clinical populations as distinct from research settings). On the basis of this, we would suggest 10 questions should be asked and separated them into those related to external validation and development (also known as derivation or discovery) samples (figure 1). The transparent reporting of research in this field is particularly important as the consequences of decisions based on some of these tools may include harm to the individual (on the basis of public safety).^10 11^

**Figure 1:**
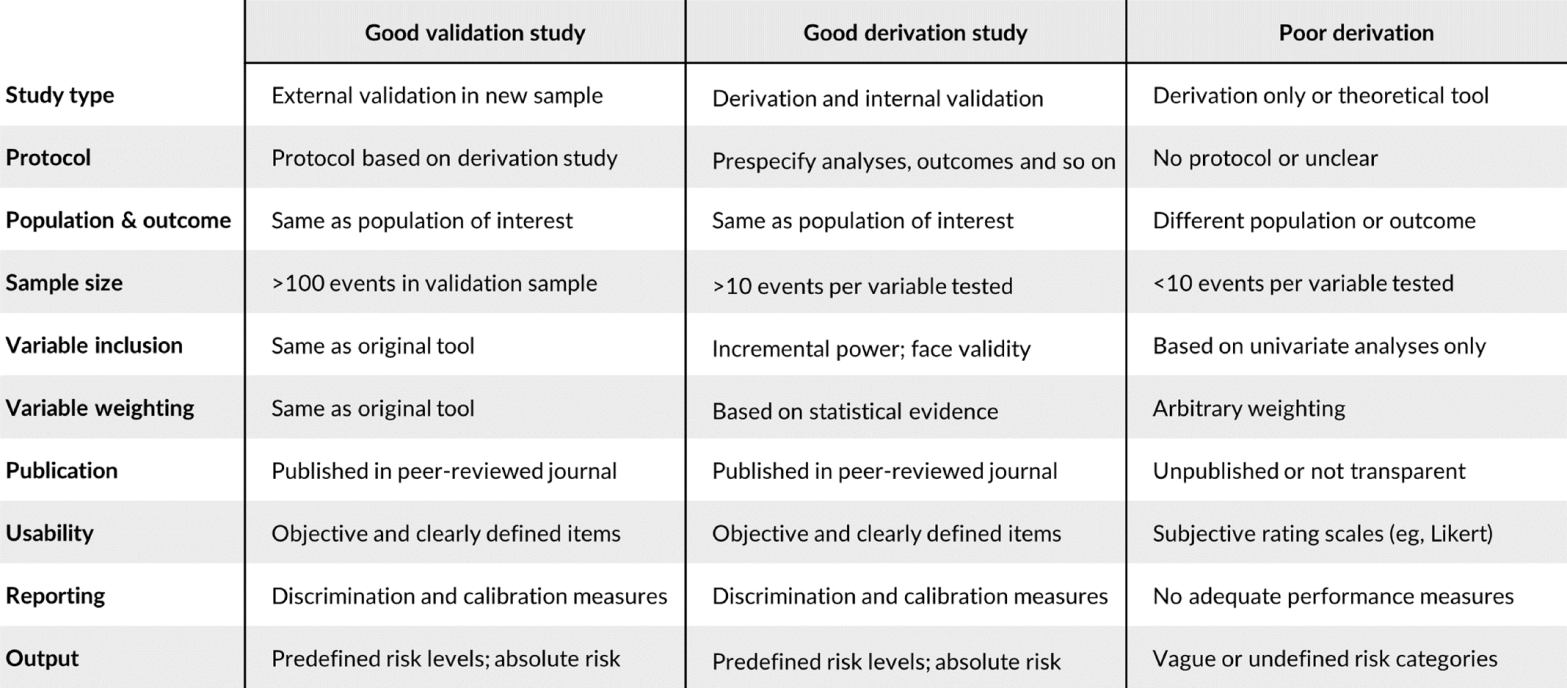
Factors to consider in determining the quality of a particular risk assessment tool.

## External validation

1. **Has the tool been externally validated?** This is the most important question. Tools tend to perform better in the sample in which they were created, as we will explain below. The only true measure of a tool’s performance is how well it predicts the outcome in a new, separate sample.^12 13^
2. **Has this validation been done in the population of interest?** The validation sample population should have similar characteristics, risk factors and baseline risk as the derivation sample, and the outcome should be the one of interest. To take two examples, if a tool is required to predict violence after release from prison, a tool validated to predict aggression in psychiatric inpatients is not appropriate. Or if the tool has only been validated in men and is used to predict risk in women, it will overestimate the risk of reoffending if sex is not adjusted for. In practice, this would explain why some tools, such as the Psychopathy Checklist, which was not developed to predict violence risk but to identify a form of personality disturbance, perform among the worse of commonly used tools in a meta-analysis.^14^ In suicide risk assessment, this would also explain why many of the currently used tools have not been found to be accurate in prospective studies as they were developed as symptom checklists (eg, Suicide Intent Scale or SAD PERSONS scale).^15^
3. **Is the tool based on sound methodology?** Risk prediction studies should be based on a protocol to avoid many of the issues described below. Validation studies should stay true to the original model and any possible changes should be prespecified in the protocol. Otherwise, the study is no longer an external validation, but the derivation of a new model. The sample size is also critical and should have at least 100 events (outcomes) for statistical power^16^ and be a representative sample for generalisability (eg, using cohort data with a high rate of non-consenting individuals will affect the study’s external validity as those who consent are likely to be different from those who do not in relation to the prevalence of background risk factors). The results should be published in peer-reviewed journals, which importantly is not in itself  a marker of methodological quality, and publications should systematically provide sufficient methodological detail to be replicable.
4. **Does it report essential information?** All tools should report both measures of *discrimination* and *calibration*. Discrimination measures the tool’s ability to distinguish between those with the outcome and those without by assigning a higher risk score or category to those who offend. This includes measures like sensitivity, specificity, positive predictive value and negative predictive value, which can be calculated at specific risk cut-offs, and area under the curve, which is an overall measure of discrimination across all possible risk cut-offs. Calibration, on the other hand, asks whether the tool predicts a risk level that is close to the observed risk. For example, a tool that predicts a 20% chance of self-harm in the high-risk group, but only 10% in the group actually self-harmed, is poorly calibrated. Calibration can be examined graphically by plotting predicted versus observed risk or through statistical tests to measure the typical level of miscalibration.
5. **Is it useful, feasible and acceptable?** The tool should provide useful information, including clearly defined risk categories, and absolute risks if appropriate. Items should also be clearly defined, objective and easy to complete. For example, subjective rating scales (eg, 1–5 Likert scales) will be difficult to rate consistently and suffer from variation between raters.^17^ The tool should have face validity by including essential items (eg, age and sex when predicting violence) and by explaining the inclusion, importance and contribution of other items. There are advantages in having interview-independent tools to mitigate against observer bias, and these have been recommended in suicide risk assessment.^18^

## Derivation (and internal validation)

If the tool has not been externally validated, it should not be routinely used in practice (apart from unusual circumstances when alternatives are not appropriate or available, and external validation is ongoing), due to important limitations. The questions below describe those limitations and how to minimise their impact and can be used to select candidate tools appropriate for external validation. Of course, even externally validated tools described above should undergo prospective validation after implementation to monitor its performance.

1. **Does the tool follow a protocol?** Again, this is a key component if the study is to provide an accurate representation of its performance. Without a protocol, the likelihood of creating a tool that reports strong performance measures, but performs poorly in practice is very high. The sample, candidate variables, outcomes, follow-up periods, statistical analyses and output should all be prespecified before any data analysis is performed.
2. **How were candidate variables selected?** Imagine a risk assessment tool based on hundreds of routinely collected variables. The analyses find that everyone who is born on a Friday has the event (eg, crime). Including this variable improves the model’s performance, but this is clearly a chance finding that will not be seen in the external sample. The more variables one tests, the more chance associations with crime will be found, and the more unrepresentative the reported model performance will be. The rule of thumb is that for each variable tested, the sample should have at least 10 outcomes.^19^ In this example, if 20 variables are tested, the sample should have at least 200 individuals committing a crime during the follow-up period to avoid the inclusion of spurious associations. In addition, the inclusion of variables should follow the protocol and involve a form of multivariable regression to determine this. Otherwise tools will include variables that do not add any incremental predictive accuracy and be more complicated and time consuming than they need to be.
3. **How are variables weighted?** Many tools give equal weighting to all included items. This assumes, first, that all included variables have the same association with the outcome, but also that the variables are all independently related to the outcome. Previous crime and income are both associated with higher risk of crime, but are they equally important? This is very unlikely to be the case, and tools that have not weighted individual items will perform worse.^20^
4. **How were other parameters selected?** If there is no protocol, and researchers find that their tool performs best at predicting the outcome at 6 months, rather than 1, 3 or 12 months, they might be tempted to use this as their primary outcome. This, however, is a form of multiple testing**—**like betting on your horse winning but, after seeing it lose, saying you were really betting on the next race. The consequence will be that the tool performs considerably worse in real-world settings.
5. **Has internal validation been done? **This method often includes bootstrapping, which takes a number of random samples from the data set to provide an estimate of accuracy of performance measures. Splitting the original sample into two random groups is a form of internal validation, as both samples will be statistically equivalent due to the randomisation. This form of splitting is occasionally presented as external validation, but it is not due to the equal distribution of predictor variables. To achieve external validation, the sample split should be based on other variables, not related to the outcome.^21^

In summary, we have provided a simple 10-point checklist that could be applied by clinicians, researchers and policy makers to test the appropriateness of a particular risk assessment tool. We recommend research investigating how many tools currently in practice meet these items, and new studies consider these issues in developing and validating new instruments across criminal justice and mental health.
